# Spironolactone Improves the All-Cause Mortality and Re-Hospitalization Rates in Acute Myocardial Infarction with Chronic Kidney Disease Patients

**DOI:** 10.3389/fphar.2021.632978

**Published:** 2021-05-31

**Authors:** Xiang Qu, Hui Yao, Changxi Chen, Shuting Kong, Lingyue Sun, Leilei Du, Siqi Liang, Zhan Gao, Gaoshu Zheng, Minghua Zheng, Chuhuan Zhao, Xiafei Feng, Gaojun Wu, Hao Zhou

**Affiliations:** ^1^ Cardiovascular Medicine, First Affiliated Hospital of Wenzhou Medical University, The Key Lab of Cardiovascular Disease of Wenzhou, Wenzhou, China; ^2^ Department of Cardiology, Ren Ji Hospital, School of Medicine, Shanghai Jiao Tong University, Shanghai, China

**Keywords:** acute myocardial infarction, spironolactone, chronic kidney disease, mortality, re-hospitalization

## Abstract

**Background:** Mineralocorticoid receptor antagonists (MRA) improve outcomes in chronic kidney disease (CKD) and acute myocardial infarction (AMI) patients. However, the lack of evidence regarding long-term clinical outcomes in the use of MRA, including spironolactone, in patients with AMI combined with CKD.

**Objectives:** This study aimed to investigate whether spironolactone could significantly reduce the risk of all-cause mortality and re-admission in patients with AMI and CKD.

**Methods:** In this single center, observational, retrospective, registry based clinical study, a total of 2,465 AMI patients were initially screened; after excluding patients with estimated glomerular filtration rate more than 60 ml/min/1.73 m^2^, 360 patients in the standard treatment group and 200 patients in the spironolactone group met the criteria. All enrolled patients follow-up for 30 months. The primary outcomes were all-cause mortality and re-admission. The key safety outcome was hyperkalemia rates during the 30 months follow-up period.

**Results:** 160 (44.4%) and 41 (20.5%) patients in the standard treatment and spironolactone groups died, respectively [hazard ratio (HR): 0.389; 95% confidence interval (CI): 0.276–0.548; *p* < 0.001]. Re-admission occurred in 217 (60.3%) and 95 (47.5%) patients in the standard treatment and spironolactone groups, respectively (HR: 0.664; 95% CI: 0.522–0.846; *p* = 0.004). The spironolactone group was divided into two based on the daily dose, low dose group (no more than 40 mg) and high dose group (more than 40 mg); the differences in the mortality rate between low dose group (16.7%) and the standard treatment group (44.4%) (HR: 0.309; 95% CI: 0.228–0.418; *p* < 0.001) and high dose group (34.1%) (HR: 0.429; 95% CI: 0.199–0.925; *p* = 0.007) were significant. The differences in re-hospitalization rate between low dose group (43.6%) and the standard treatment group (60.3%) (HR: 0.583; 95% CI: 0.457–0.744; *p* < 0.001) and high dose group (61.4%) (HR: 0.551; 95% CI: 0.326–0.930; *p* = 0.007) was significant. Hyperkalemia occurred in 18 (9.0%) and 18 (5.0%) patients in the spironolactone group and standard treatment group, respectively (HR: 1.879; 95% CI: 0.954–3.700; *p* = 0.068). Whereas, Hyperkalemia occurred in high dose group (20.5%) significantly more often than in the standard treatment group (*p* < 0.001) and low dose group (5.8%) (*p* = 0.003).

**Conclusion:** Using MRA, such as spironolactone, may substantially reduce the risk of both all-cause mortality and re-admission in patients with AMI and CKD; the use of low-dose spironolactone has the best efficacy and safety. However, this was a relatively small sample size, single center, observational, retrospective, registry based clinical study and further prospective evaluation in adequately powered randomized trials were needed before further use of spironolactone in AMI with CKD population.

**Clinical Trial Registration:** chictr.org.cn, identifier ChiCTR1900021344

## Introduction

Chronic kidney disease (CKD) affects approximately 30–40% of all patients with acute myocardial infarction (AMI) (Fox et al., 2010) and has been associated with a particularly high risk of adverse cardiovascular outcomes (Anavekar et al., 2004); in these patients, the age-adjusted mortality is 15- to 30-fold higher than that in the general population (Go et al., 2004; Tonelli et al., 2006). Large-scale trials have shown that medical therapy targeting renin-angiotensin-aldosterone system, including angiotensin-converting enzyme inhibitors (ACEIs) and angiotensin receptor blockers (ARBs), is beneficial against adverse cardiovascular outcomes in the general population (Evans et al., 2016; Korhonen et al., 2017). However, data supporting the positive effects of mineralocorticoid receptor antagonists (MRA) as a renin-angiotensin-aldosterone system-targeting therapy are limited for cardiovascular outcomes in patients with CKD and AMI because these patients are often excluded from most clinical trials on which guidelines are based (Coca et al., 2006). However, recent studies suggest that the protective mechanisms of MRA may be qualitative or quantitative changes in aldosterone rather than angiotensin II (Struthers and MacDonald, 2004).

High aldosterone plasma levels early after AMI are related to mortality, sudden cardiac death, and heart failure (Beygui et al., 2009). Several experimental studies and clinical trials have confirmed that early MRA administration after myocardial infarction could improve myocardial structure, electrical remodeling, cardiovascular fibrosis, inflammation, oxidative stress, and reduce mortality (Pitt et al., 2005; Kasama et al., 2011; Beygui et al., 2016; Bulluck et al., 2019). In patients with CKD, MRA have been shown to slow renal progression by reducing proteinuria (Bianchi et al., 2006), as well as reduce cardiovascular morbidity and mortality (Matsumoto et al., 2014).

However, the lack of evidence for MRA, including spironolactone, in patients with AMI and CKD, especially regarding long-term clinical outcomes, such as all-cause mortality, have limited their clinical application. On this basis, in this study we aimed to investigate whether spironolactone could significantly reduce the risk of mortality from all causes and re-hospitalization in patients with AMI and CKD.

## Methods

### Study Population

This was a single center, observational, retrospective, registry based clinical study. The purpose of this study was to assess whether a spironolactone regimen in patients with AMI and CKD reduced long-term all-cause mortality and re-admission rates. Data from consecutive patients between January 2013 and December 2017 were collected. We enrolled AMI patients undergoing emergency treatment with percutaneous coronary intervention (PCI) at the First Affiliated Hospital of Wenzhou Medical University where treatment for acute cardiac disease is provided. Patients with a diagnosis of AMI were given aspirin, clopidogrel, or ticagrelor loading dose immediately before PCI. Dual antiplatelet therapy was routine used after PCI. Statins, β-blockers or ACEI/ARB or MRA were administered to improve myocardial remodeling after contraindications were excluded. According to whether they were treated with spironolactone after PCI, the patients were divided into two groups: standard treatment group and spironolactone group. Patients in spironolactone regimen received oral spironolactone as soon as possible. The first oral spironolactone was administered in 24 h after PCI, after control of plasma concentrations of potassium. The oral dose was not given if the first blood sample revealed a potassium level more than 5.5 mmol/L. Furthermore, the spironolactone group was then divided into two groups according to their treatment dose, patients took no more than 40 mg per day as low dose group and patients took more than 40 mg per day as high dose group; subgroup analysis was then performed. Patients were received spironolactone during the 30 months of follow-up unless adverse events such as recurrent hyperkalemia occurred. Only the first myocardial infarction during the specified period was considered. A diagnosis of AMI was made according to the universal definition of AMI (detection of a rise in cardiac biomarker, such as troponin, values with at least one value above the 99th percentile upper reference limit and with at least one of the following: symptoms of ischemia, new ST-segment–T wave changes, new left bundle branch block, or pathological Q waves in the electrocardiogram, imaging evidence of new loss of viable myocardium, or intracoronary thrombus on angiography) (Thygesen et al., 2012). The study protocol was approved by the Ethics Review Board of the First Affiliated Hospital of Wenzhou Medical University. Clinical trial registration at chictr.org.cn and the identifier was ChiCTR1900021344.

### Renal Function Assessment

The estimated glomerular filtration rate (eGFR) was used to assess patients’ renal function. The eGFR was calculated from the creatinine level on admission using the Chronic Kidney Disease Epidemiology Collaboration equation (CKD-EPI) (Levey et al., 2007). The creatinine level measurements were performed by either an enzymatic or corrected Jaffe method (alkaline picrate reaction), both of which are comparable to isotope dilution mass spectrometry standards. Renal function was graded based on eGFR using the Kidney Disease Improving Global Outcomes 2012 Guideline definition (Summary of recommendation statements, 2013). A total of 2465 AMI patients were initially screened and 2,420 had available eGFR data. Among the patients with an identified eGFR, 560 had moderate to severe CKD with eGFR < 60 ml/min/1.73 m^2^ based on the definition of CKD.

### Data Collection

The data were collected from the hospital database using a web-based electronic medical record system, based on inpatient and outpatient follow-up results. The following demographic and clinical characteristics were extracted: age, sex, body mass index, heart rate, pulsed blood pressure (systolic blood pressure minus diastolic blood pressure), and traditional cardiovascular risk factors including smoking, alcohol intake, hypertension, diabetes, coronary heart disease, and stroke. Key laboratory data considered as prognostic markers were also collected, including eGFR (calculated based on serum creatinine), alanine aminotransferase, aspartate aminotransferase, low-density lipoprotein cholesterol, brain natriuretic peptide (BNP), troponin I (cTnI), D-dimer, hemoglobin, and potassium levels. Echocardiography measurements, including the left ventricular ejection fraction (LVEF) as calculated based on four apical four- and two-chamber views using the modified Simpson’s biplane method, left atrial diameter using the M-mode echocardiography in the anteroposterior left atrial dimension of parasternal, and left ventricular end diastolic diameter. Post-PCI medications, such as β-blockers, ACEIs/ABRs, statins, and spironolactone, as well as their dosage, were also identified.

### Follow-Up and Outcomes

We examined outcome data in medical records, reviews, and telephone correspondence within 30 months after PCI. The primary outcomes were all-cause mortality and re-hospitalization. Serum potassium levels were also collected during subsequent follow-up and more than 5.5 mmol/L was considered hyperkalemia.

### Statistical Analysis

All categorical data are shown as frequencies and percentages. The statistics for continuous variables are expressed as means and standard deviations or as median and interquartile range when the distribution was not normal. Categorical variables as frequencies and proportions. Baseline characteristics were compared between the two groups. A Fisher’s exact test or chi-square test was used to identify differences in categorical data, and independent-sample t test (data with normal distribution) or Mann–Whitney U test (data with non-normal distribution) were used to identify differences in quantitative data.

Kaplan-Meier curves were drawn to visualize all-cause mortality and re-hospitalization distribution over time for patients who did and did not take spironolactone after PCI, and a comparison between the two groups was made using a Cox-Mantel test. In addition, univariate followed by multivariate Cox proportional hazards regression analyses were constructed to determine the risk factors for outcomes after adjusting for individual risk factors. Factors found to have predictive significance (*p* < 0.10) in the univariate analysis and baseline clinical variables with significant differences (*p* < 0.05) between the two groups were included in the multivariate regression model. In Cox-Mantel test, alanine aminotransferase, aspartate aminotransferase, BNP, and cTnI data underwent natural log transformation as the data were large numerical range. At last, the consistency of the treatment effect on the two main outcomes was assessed among subgroups including: age, sex, body mass index, LVEF, left ventricular end diastolic diameter, eGFR, low-density lipoprotein cholesterol level, potassium level, history of hypertension, diabetes mellitus, smoking, alcohol use, and use of β-blockers, ACEIs/ABRs, statins. The effect in each subgroup was analyzed with the use of an unadjusted Cox model, and *p*-values for the interaction were calculated using a Cox proportional hazards regression model. Hazard ratios (HRs), 95% confidence intervals (CIs), and *p*-values were calculated. All analyses were performed using SPSS software version 26 (SPSS, Inc. Chicago, IL). *p*-values < 0.05 were considered statistically significant.

## Results

### Study Group Characteristics

**FIGURE 1:**
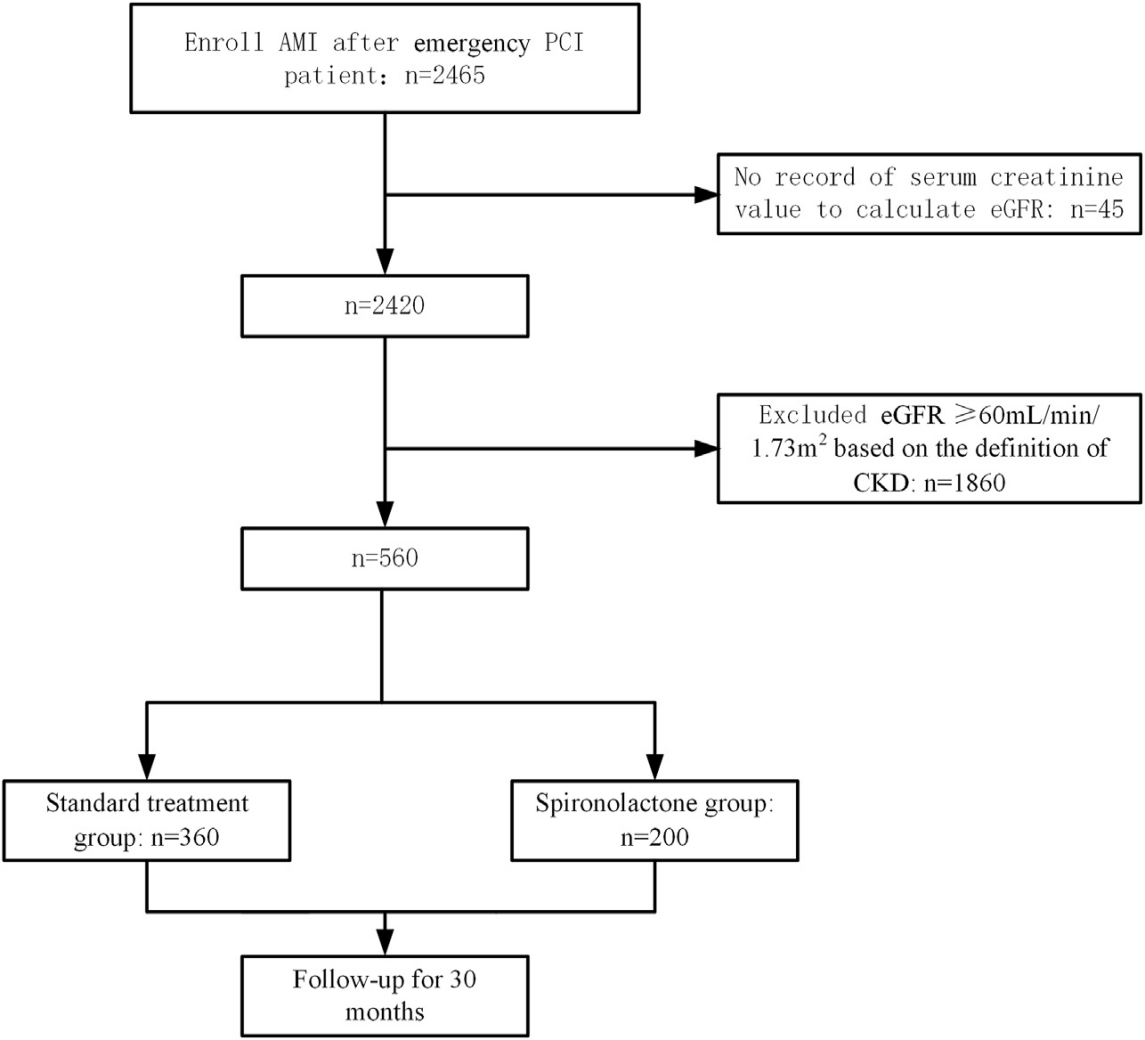
Patient enrollment and follow-up flow diagram. AMI, acute myocardial infarction; PCI, percutaneous coronary intervention; eGFR, estimated glomerular filtration rate; CKD, chronic kidney disease.

The study profile is presented in Figure 1. The patients’ baseline characteristics are presented in Table 1. Overall, from the initial 2,465 patients who underwent emergency PCI, 360 patients in the standard treatment group and 200 patients in the spironolactone group in the current analysis. The two groups were significantly different in several variables; the spironolactone group had a lower level of potassium, D-dimer and LVEF, but had a higher level of age, eGFR and left ventricular end diastolic diameter and usage of β-blockers, ACEIs/ABRs and statins.

**TABLE 1:** Baseline clinical characteristics of patients.

Clinical parameters	Standard treatment group (n = 360)	Spironolactone group (n = 200)	*p* Value
Demographics			
Age (y)	71.99 ± 10.58	73.94 ± 9.46	0.031*
Male sex, n (%)	260 (72.2)	129 (64.5)	0.057
Body mass index (kg/m^2^)	23.88 ± 3.52	23.85 ± 3.14	0.916
Heart rate (bpm)	85.76 ± 22.44	84.58 ± 20.73	0.539
Pulsed blood pressure (mmHg)	49.84 ± 20.56	48.52 ± 18.31	0.450
Laboratory measurements			
Alanine aminotransferase (U/L)	54.00 (33.00–98.50)	58.00 (31.00–109.50)	0.464
Aspartate aminotransferase (U/L)	232.00 (99.00–444.00)	277.50 (122.00–299.50)	0.375
eGFR (ml/min/1.73 m^2^)	35.56 ± 16.55	41.48 ± 13.26	<0.001*
Low-density lipoprotein cholesterol level (mmol/L)	2.91 ± 1.14	2.91 ± 1.06	0.970
BNP (pg/ml)	236.00 (76.00–768.00)	439.50 (123.75–979.25)	0.085
cTnI (ng/ml)	17.73 (1.34–51.51)	33.21 (5.35–49.96)	0.686
D-dimer (mg/L)	3.30 ± 4.38	2.41 ± 3.59	0.021*
Hemoglobin (g/L)	119.64 ± 21.40	122.72 ± 18.13	0.087
Potassium levels (mmol/L)	4.37 ± 0.75	4.20 ± 0.60	0.006*
Medical history, n (%)			
Diabetes mellitus	109 (30.3)	61 (30.5)	0.956
Hypertension	246 (68.3)	138 (69.0)	0.871
Smoking	123 (34.2)	55 (27.5)	0.105
Alcohol use	67 (18.6)	27 (13.5)	0.121
Coronary heart disease	30 (8.3)	16 (8.0)	0.891
Stroke	34 (9.4)	20 (10.0)	0.831
Echocardiography measurements			
LVEF (%)	47.72 ± 10.70	43.81 ± 9.41	<0.001*
Left atrial diameter (mm)	39.98 ± 5.01	40.63 ± 5.80	0.214
left Ventricular	50.00 ± 6.41	51.25 ± 7.35	0.038*
End diastolic diameter (mm)			
Medications, n (%)			
β-blockers	148 (41.1)	119 (59.5)	<0.001*
ACEIs/ABRs	114 (31.7)	108 (54.0)	<0.001*
Statins	274 (76.1)	181 (90.5)	<0.001*

Data are mean ± SD or number (%). eGFR, estimated glomerular filtration rate; BNP, brain natriuretic peptide; cTnI, troponin I; LVEF, left ventricular ejection fraction; ACEIs, angiotensin-converting enzyme inhibitors; ARBs, angiotensin receptor blockers.

* *p* < 0.05.

### Long-Term Clinical Primary Outcomes in AMI Patients With CKD

**FIGURE 2:**
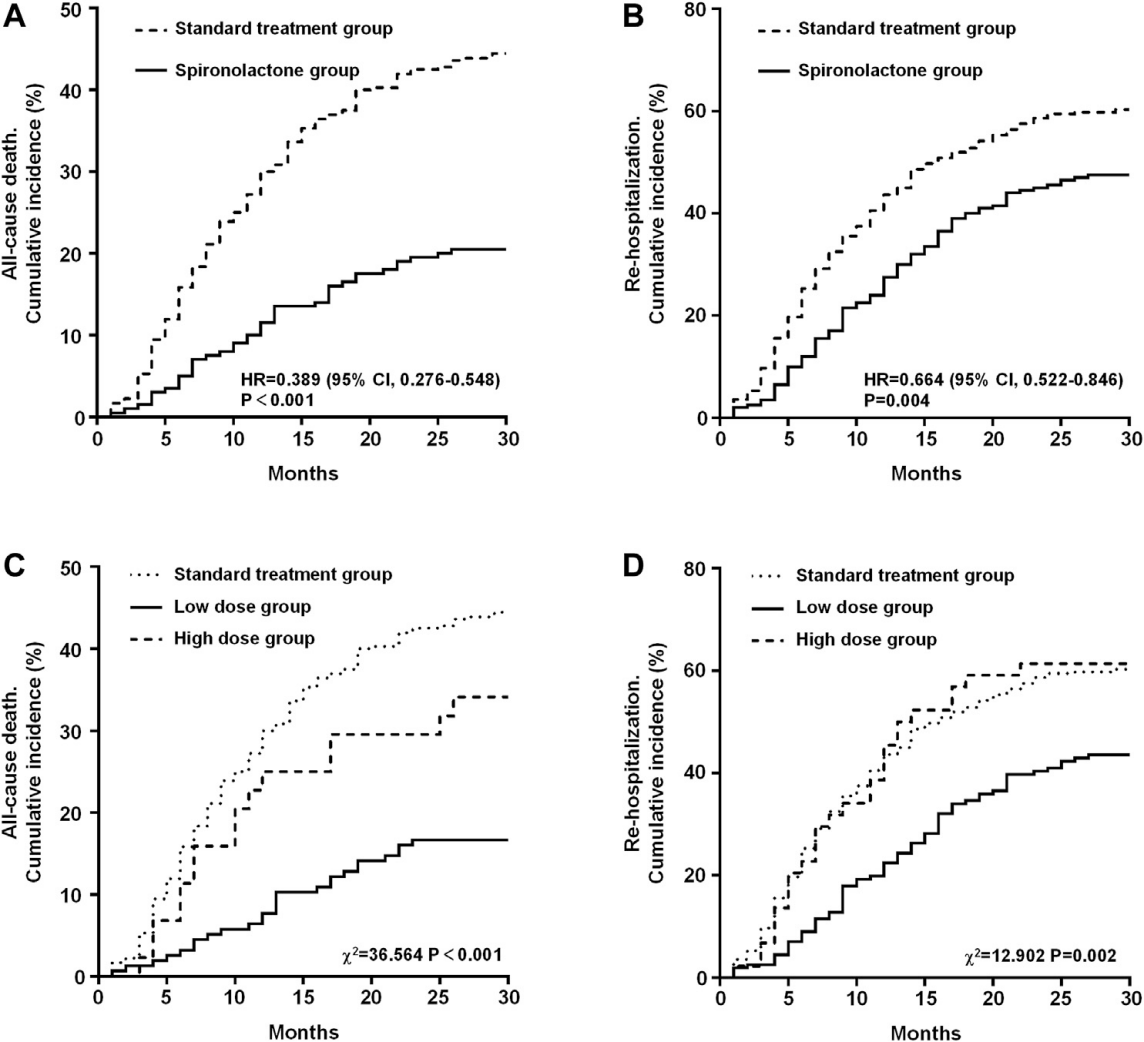
Kaplan-Meier estimates in the standard treatment group and spironolactone group for the rate of all-cause mortality **(A)** and re-hospitalization **(B)**. Kaplan-Meier estimates of all-cause mortality **(C)** and re-hospitalization rates **(D)** grouped by dose of spironolactone. CI, confidence interval; HR, hazard risk.

The primary outcomes in the standard treatment group and spironolactone group are given in Table 2. Compared with those with standard treatment, spironolactone significantly reduced the risk for all-cause death and re-hospitalization. A total of 201 (35.9%) patients died, comprising 160 (44.4%) receiving standard treatment and 41 (20.5%) treated with spironolactone [hazard ratio (HR): 0.389; 95% confidence interval (CI): 0.276–0.548; *p* < 0.001] (Figure 2A). In total, 312 (55.7%) patients were readmitted; 217 (60.3%) were in the standard treatment group and 95 (47.5%) patients were in the spironolactone group (HR: 0.664; 95% CI: 0.522–0.846; *p* = 0.004) (Figure 2B).

**TABLE 2:** Primary outcome after follow-up.

Primary outcome	Spironolactone group (n = 200)	Standard treatment group (n = 360)	Hazard Ratio (95% CI)	*p* Value
All-cause mortality, n (%)	41 (20.5%)	160 (44.4%)	0.389 (0.276–0.548)	<0.001
Re-hospitalization, n (%)	95 (47.5%)	217 (60.3%)	0.664 (0.522–0.846)	0.004

CI, confidence interval.

The results of the long-term clinical primary outcome analyses grouped by daily dose of spironolactone (standard treatment group, low dose group, and high dose group) are shown in Figure 2. After grouping, there were 360 patients in the standard treatment group, 156 patients in low dose group and 44 patients in high dose group. The all-cause death rate in the standard treatment group, low dose group, and high dose group were 44.4% (n = 160), 16.7% (n = 26), and 34.1% (n = 15), respectively. Mortality was significantly different between the three groups (χ^2^ = 36.564, *p* < 0.001). On post-hoc analysis, the difference in mortality between low dose group and the standard treatment group (HR: 0.309; 95% CI: 0.228–0.418; *p* < 0.001) and high dose group (HR: 0.429; 95% CI: 0.199–0.925; *p* = 0.007) was significant. But there was no significant difference between high dose group and standard treatment group (HR: 0.713; 95% CI: 0.449–1.131; *p* = 0.201) (Figure 2C). In addition, the re-hospitalization rate in the standard treatment group, low dose group, and high dose group were 60.3% (n = 217), 43.6% (n = 68), and 61.4% (n = 27), respectively, and was significantly different between the three groups (χ^2^ = 12.902, *p* = 0.002). On post-hoc analysis, the difference in re-hospitalization rate between low dose group and the standard treatment group (HR: 0.583; 95% CI: 0.457–0.744; *p* < 0.001) and high dose group (HR: 0.551; 95% CI: 0.326–0.930; *p* = 0.007) was significant. Also, there was no significant difference between high dose group and standard treatment group (HR: 1.024; 95% CI: 0.684–1.534; *p* = 0.905) (Figure 2D).

### Subgroup and Multivariate Analysis of Primary Outcomes in AMI with CKD Patients

**FIGURE 3:**
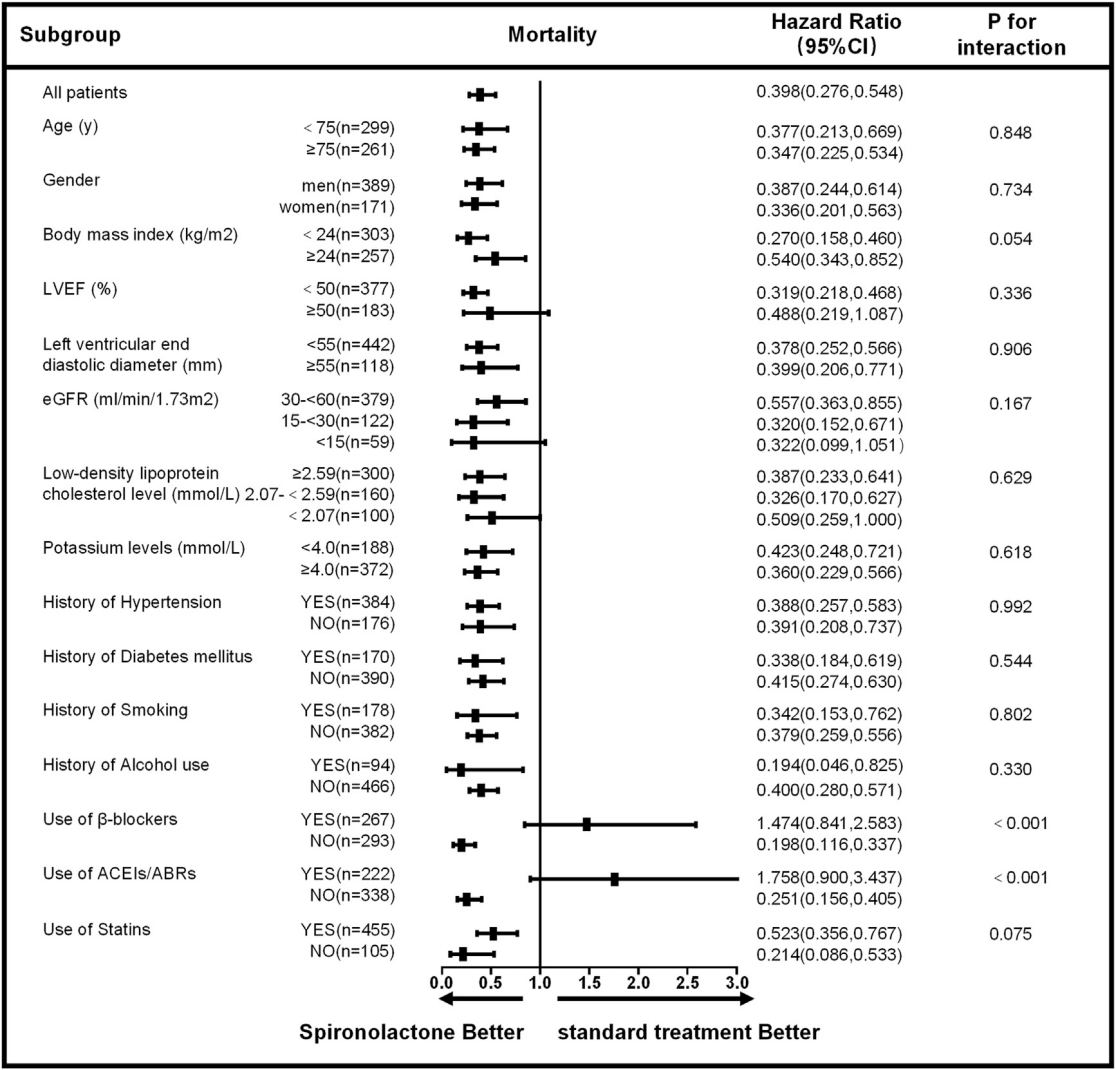
Subgroup analysis of all-cause mortality based on the long-term follow-up. LVEF, left ventricular ejection fraction; eGFR, estimated glomerular filtration rate; ACEIs, angiotensin-converting enzyme inhibitors; ARBs, angiotensin receptor blockers; CI, confidence interval.

**FIGURE 4:**
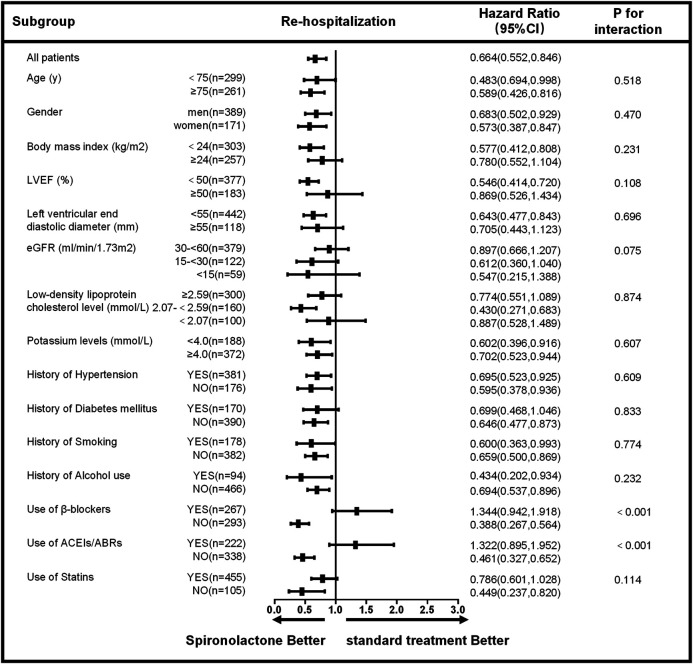
Subgroup analysis of re-hospitalization based on long-term follow-up. LVEF, left ventricular ejection fraction; eGFR, estimated glomerular filtration rate; ACEIs, angiotensin-converting enzyme inhibitors; ARBs, angiotensin receptor blockers; CI, confidence interval.

The subgroup analysis for all-cause mortality and re-admission (by age, sex, body mass index, LVEF, left ventricular end diastolic diameter, eGFR, low-density lipoprotein cholesterol level, potassium level, the history of hypertension, diabetes mellitus, smoking, alcohol use and the use of β-blockers, ACEIs/ABRs, and statins) is given in Figures 3, 4. The reduction in the risk of the primary outcomes in the spironolactone group was consistent among subgroups. Considering mortality and re-admission, a significant interaction was found between the use of spironolactone and use of ACEIs/ARBs (*p* < 0.001) or β-blockers (*p* < 0.001). In addition, Spironolactone significantly increased serum potassium in patients using ACEI/ARB (standard treatment: 4.64 ± 0.46 mmol/L vs spironolactone: 5.04 ± 0.62 mmol/L; *p* < 0.001) or β-blocker (standard treatment: 4.69 ± 0.60 mmol/L vs spironolactone: 4.94 ± 0.45 mmol/L; *p* = 0.011).

Based on the univariate analysis, the following factors were associated with all-cause mortality in patients with AMI and CKD: use of spironolactone (HR: 0.389; 95% CI: 0.276–0.548; *p* < 0.001), use of β-blocker, ACEIs/ABRs or statins, age, gender, heart rate, Ln alanine aminotransferase, Ln aspartate aminotransferase, CKD stage, low-density lipoprotein cholesterol, LnBNP, D-dimer, hemoglobin, potassium, history of smoking, alcohol use, coronary heart disease, and LVEF. After adjusting for confounding factors, the Cox regression analysis showed the use of spironolactone was independently associated with a 47% reduction in risk of all-cause death in patients with AMI and CKD (HR: 0.530; 95% CI: 0.339–0.828; *p* = 0.005). Younger age, higher LVEF, and use of β-blockers, ACEIs/ABRs and statins were also significant independent negative predictors of all-cause mortality; CKD stage 5 and history of coronary heart disease were independent positive predictors (Table 3). In addition, the use of spironolactone (HR: 0.664; 95% CI: 0.522–0.846; *p* = 0.004), use of β-blocker, ACEIs/ABRs or statins, age, gender, heart rate, Ln alanine aminotransferase, Ln aspartate aminotransferase, CKD stage, low-density lipoprotein cholesterol, LnBNP, D-dimer, hemoglobin, potassium, history of diabetes, smoking, alcohol use, coronary heart disease and LVEF. were also associated with re-admission in the univariate analysis. In the Cox regression analysis, the use of spironolactone was independently associated with a 27.1% reduced risk of re-admission in patients with AMI and CKD (HR: 0.729; 95% CI: 0.533 to 0.997; *p* = 0.048). Younger age, male, higher LVEF and use of statins were also significant independent negative predictors of re-hospitalization and higher CKD level, higher LnBNP and left ventricular end diastolic diameter and history of diabetes mellitus were independent positive predictor (Table 4).

**TABLE 3:** Independent predictors for mortality in AMI patients with CKD.

	Univariate			Multivariate		
	HR	95% CI	*p* Value	HR	95% CI	*p* Value
Age (y)	1.031	1.015–1.046	<0.001	1.040	1.017–1.064	<0.001*
Male sex, n (%)	0.606	0.457–0.804	0.001	0.811	0.521–1.263	0.355
Body mass index (kg/m^2^)	1.003	0.963–1.046	0.870			
Heart rate (bpm)	1.004	1.008–1.020	<0.001	1.005	0.996–1.014	0.252
Pulsed blood pressure (mmHg)	0.994	0.987–1.002	0.152			
Ln alanine aminotransferase	1.234	1.082–1.407	0.002	1.030	0.737–1.440	0.861
Ln aspartate aminotransferase	1.180	1.030–1.353	0.017	1.154	0.843–1.578	0.372
CKD Stage 3		1			1	
CKD Stage 4	2.647	1.934–3.624	<0.001	1.482	0.936–2.346	0.093
CKD Stage 5	4.202	2.876–6.140	<0.001	2.721	1.541–4.803	0.001*
Low-density lipoprotein cholesterol level (mmol/L)	0.873	0.750–1.017	0.081	0.920	0.773–1.095	0.348
Ln BNP	1.192	1.315–1.451	<0.001	1.177	1.003–1.382	0.046*
Ln cTnI	1.012	0.950–1.079	0.711			
D-dimer (mg/L)	1.069	1.041–1.099	<0.001	1.005	0.958–1.055	0.825
Hemoglobin (g/L)	0.987	0.981–0.993	<0.001	0.999	0.989–1.010	0.907
Potassium levels (mmol/L)	1.340	1.101–1.631	0.004	0.976	0.734–1.299	0.869
History of diabetes mellitus	1.248	0.932–1.672	0.137			
History of hypertension	1.091	0.806–1.475	0.574			
History of smoking	1.270	1.080–1.493	0.004	1.120	0.662–1.896	0.672
History of alcohol use	0.628	0.410–0.962	0.033	1.007	0.536–1.894	0.982
History of coronary heart disease	1.505	0.966–2.343	0.071	2.193	1.244–3.866	0.007*
History of stroke	1.322	0.863–2.025	0.199			
LVEF (%)	0.955	0.940–0.971	<0.001	0.975	0.954–0.996	0.019*
Left atrial diameter (mm)	1.008	0.978–1.040	0.602			
Left ventricular end diastolic diameter (mm)	1.005	0.982–1.028	0.679	1.015	0.985–1.045	0.325
Use of β-blockers	0.277	0.201–0.383	<0.001	0.503	0.334–0.757	0.001*
Use of ACEIs/ABRs	0.268	0.187–0.385	<0.001	0.612	0.390–0.961	0.033*
Use of statins	0.314	0.235–0.421	<0.001	0.583	0.375–0.907	0.017*
Use of spironolactone	0.389	0.276–0.548	<0.001	0.530	0.339–0.828	0.005*

Significance variables in the univariate analysis (*p* < 0.10) were included in the multivariate regression model. Abbreviations: AMI, acute myocardial infarction; CKD, chronic kidney disease; BNP, brain natriuretic peptide; cTnI, troponin I; LVEF, left ventricular ejection fraction; ACEIs, angiotensin-converting enzyme inhibitors; ARBs, angiotensin receptor blockers; HR, Hazard risk; CI, confidence interval.

* *p* < 0.05.

**TABLE 4:** Independent predictors for Re-hospitalization in AMI patients with CKD.

	Univariate			Multivariate		
	HR	95% CI	*p* Value	HR	95% CI	*p* Value
Age (y)	1.022	1.010–1.034	<0.001	1.035	1.017–1.053	<0.001*
Male sex, n (%)	0.669	0.531–0.843	0.001	0.640	0.451–0.908	0.013*
Body mass index (kg/m2)	0.993	0.961–1.027	0.690			
Heart rate (bpm)	1.010	1.005–1.015	<0.001	1.002	0.995–1.008	0.635
Pulsed blood pressure (mmHg)	0.996	0.990–1.002	0.207			
Ln alanine aminotransferase	1.217	1.089–1.359	0.001	0.972	0.742–1.273	0.834
Ln aspartate aminotransferase	1.109	0.994–1.236	0.064	1.171	0.921–1.489	0.198
CKD Stage 3		1			1	
Stage 4	2.356	1.820–3.050	<0.001	1.530	1.060–2.209	0.023*
Stage 5	3.667	2.638–5.097	<0.001	3.478	2.187–5.532	<0.001*
Low-density lipoprotein cholesterol level (mmol/L)	0.854	0.758–0.963	0.010	0.869	0.754–1.001	0.052
LnBNP	1.288	1.190–1.393	<0.001	1.129	1.005–1.269	0.040*
LncTnI	1.001	0.951–1.053	0.979			
D-dimer (mg/L)	1.073	1.049–1.097	<0.001	1.030	0.994–1.068	0.100
Hemoglobin (g/L)	0.989	0.984–0.994	<0.001	0.999	0.991–1.008	0.886
Potassium levels (mmol/L)	1.432	1.216–1.686	<0.001	1.142	0.913–1.428	0.244
History of diabetes mellitus	1.429	1.132–1.804	0.003	1.451	1.059–1.987	0.020*
History of hypertension	1.170	0.918–1.491	0.205			
History of smoking	1.491	1.160–1.917	0.002	0.666	1.089–0.738	1.607
History of alcohol use	0.755	0.549–1.039	0.084	1.066	0.677–1.680	0.783
History of coronary heart disease	1.377	0.950–1.995	0.091	1.496	0.946–2.368	0.085
History of stroke	1.137	0.793–1.632	0.485			
LVEF (%)	0.968	0.956–0.980	<0.001	0.974	0.958–0.990	0.002*
Left atrial diameter (mm)	1.015	0.990–1.039	0.240			
Left ventricular end diastolic diameter (mm)	1.014	0.996–1.031	0.122	1.026	1.003–1.050	0.027*
Use of β-blockers	0.568	0.452–0.713	<0.001	0.852	0.635–1.141	0.282
Use of ACEIs/ABRs	0.615	0.485–0.779	<0.001	1.012	0.744–1.376	0.941
Use of statins	0.478	0.370–0.616	<0.001	0.654	0.462–0.926	0.017*
Use of spironolactone	0.664	0.522–0.846	0.001	0.729	0.533–0.997	0.048*

Significance variables in the univariate analysis (*p* < 0.10) were included in the multivariate regression model. Abbreviations as in Table 3.

* *p* < 0.05.

### Safety

Hyperkalemia occurred in 18 (9.0%) and 18 (5.0%) patients in the spironolactone group and standard treatment group, respectively (HR: 1.879; 95% CI: 0.954–3.700; *p* = 0.068). Furthermore, the spironolactone group was divided by daily dose; nine (5.8%) patients in low dose group and nine (20.5%) patients in high dose group developed hyperkalemia. Hyperkalemia occurred in high dose group significantly more often than in the standard treatment group (*p* < 0.001) and low dose group (*p* = 0.003). There was no significant difference between the standard treatment group and low dose group (*p* = 0.719). After grouped by eGFR, hyperkalemia occurred in 19 (5.0%), 12 (9.8%) and 5 (8.5%) patients in CKD stage 3, 4 and 5 groups, respectively. There was no statistically significant difference among the three groups (*p* = 0.133). The change of serum potassium after use spironolactone was also no statistically significant difference among CKD stage 3, 4 and 5 groups (0.85 ± 0.68 mmol/L vs 0.93 ± 0.64 mmol/L vs 1.08 ± 0.67 mmol/L; *p* = 0.246).

In the spironolactone group, there were five patients in low dose spironolactone group and six patients in high dose spironolactone group discontinued taking spironolactone because of recurrent hyperkalemia. Three patients in high dose spironolactone group had occurred life-threatening hyperkalemia such as malignant arrhythmia and dialysis was required to lower serum potassium levels. There were 1 (11.1%) patient in low dose spironolactone group and 2 (22.2%) patients in high dose spironolactone group had severe hyperkalemia but no statistical difference between two groups (*p* = 0.527).

## Discussion

Based on the results from this cohort clinical registry trial, 22.7% of patients with AMI had concomitant moderate to severe CKD with an eGFR < 60 ml/min/1.73 m^2^. A total of 560 patients were included in the current analysis, 35.7% of whom received spironolactone. Although MRA are proven beneficial against adverse cardiovascular outcomes, whether the use of spironolactone confers a benefit in AMI patients with CKD is questionable. We showed that treatment with spironolactone was associated with a 47% lower risk of death and 27.1% lower risk of re-admission as compared with those following standard treatment and the benefit remained in various subgroups (Figures 3, 4). In addition, spironolactone daily doses of ≤ 40 mg had better clinical primary outcomes (Figure 2). Notably, AMI patients with an eGFR <60 ml/min/1.73 m^2^ taking > 40 mg spironolactone per day did not benefit from spironolactone use.

Elevated aldosterone levels in AMI patient have been shown to correlate with worse adverse clinical outcomes, including mortality (Beygui et al., 2006). Therefore, MRA have been used to improve short- and long-term clinical outcomes in patients who have AMI. Previous studies, such as the RALES trial (Pitt et al., 1999) and EMPHASIS–HF (Zannad et al., 2011) trial, have attributed this reduction to improved heart failure after AMI. In the EMPHASIS–HF trial, patients with eGFR < 30 ml/min/1.73 m^2^ were excluded and renal insufficiency patients were only analyzed in the subgroup analysis. Whereas, in our study, we focused on the long-term clinical outcomes of spironolactone in patients with renal insufficiency (including eGFR <30 ml/min/1.73 m^2^). For patients with AMI without heart failure, such as in the REMINDER trial (Montalescot et al., 2014), ALBATROSS trial (Beygui et al., 2016) and MINIMIZE STEMI trial (Bulluck et al., 2019), the application of MRA reduced BNP levels, mortality, and size of ST-segment elevation myocardial infarction. Recently, two large meta-analyses have also shown the benefits of MRA in reducing mortality and re-admission in post-myocardial infarction patients with or without heart failure (Bossard et al., 2018; Dahal et al., 2018). Moreover, MRA-based treatment models to reduce cardiovascular event risk in patients with CKD have been gaining increasing interest. The risk factors for cardiovascular disease in CKD patients was significantly different from those of the general population, specifically, raised plasma aldosterone levels. In heart failure with preserved ejection fraction (HFpEF) with CKD patients, the efficacy of spironolactone is still unclear. In HFpEF patients, Pitt B et al. (Pitt et al., 2014) discovered that treatment with spironolactone did not significantly reduce the incidence of death from cardiovascular causes or hospitalization for the management of heart failure. Whereas, Beldhuis IE et al. (Beldhuis et al., 2019) analyzed data from HFpEF patients enrolled in the TOPCAT Americas study and found consistent efficacy of spironolactone was observed in decreased eGFR. These data supported use of spironolactone to treat HFpEF with CKD patients. Data derived from the DOHAS trial (Matsumoto et al., 2014) have confirmed that spironolactone leads to substantive benefits in both death from all causes and re-admission, even in hemodialysis patients. Therefore, high plasma aldosterone is recognized as a common risk factor for adverse cardiovascular events in CKD and AMI patients. However, the influence of MRA therapy on clinical outcomes in AMI patients with CKD is less well established. As a consequence, our study was important as it was the first to assess whether MRA therapy provided a benefit against long-term all-cause mortality and re-hospitalization. Furthermore, different dosages of spironolactone were preliminarily explored in subgroups in order to determine which daily dose of spironolactone is the optimal choice for long-term clinical outcomes in AMI with CKD patients.

For patients with AMI and CKD, the optimal dosing of spironolactone remains to be established. Our study found that the low-dose spironolactone group had the best long-term prognosis among these patients. A previous paper also had noted that low-dose spironolactone treatment (≤40 mg per day) of patients with CKD and an eGFR < 60 ml/min/1.73 m^2^ tended to reduce all-cause death and cardiogenic re-admission rather than among those with an eGFR > 60 ml/min/1.73 m^2^ (Vardeny et al., 2012). Even in hemodialysis patients, low-dose spironolactone was also associated with reduced cardiovascular mortality (Matsumoto et al., 2014). Low-dose spironolactone is considered beneficial for left ventricular remodeling in patients with preserved cardiac function after AMI, although no studies have compared the mortality and re-admission of AMI patients given different doses of spironolactone (Vatankulu et al., 2013; Bulluck et al., 2019). David et al. found that with increased spironolactone dosages ( > 40 mg per day), the rate of potential adverse effects, especially hyperkalemia, also increased. Simultaneously, the all-cause mortality rate and re-hospitalization rate increased by a factor of about three. Among them, the rates for hyperkalemia-associated hospital admission and hyperkalemia-associated death were nearly doubled (Juurlink et al., 2004). Our study discovered high dose group had higher rate of hyperkalemia and further found that a high dose of spironolactone may offset the benefit of low dose of spironolactone, resulting in a long-term clinical outcome comparable to that of patients who did not take spironolactone.

Hyperkalemia is a well-recognized adverse outcome in patients treated with MRA. Previous research also shows that 44% of patients who experienced hyperkalemia were on a high daily dose of spironolactone (>50 mg per day) (Wei et al., 2010). In a study on the safety of spironolactone for regular hemodialysis, 32% of cases of hyperkalemia were associated with high doses of spironolactone (>50 mg per day) (Charytan et al., 2019).

In our research, the risk for hyperkalemia in patients with high dose of spironolactone was significantly increased. Thus, we considered closer laboratory monitoring of serum potassium and more judicious use of spironolactone in both inpatient and outpatient settings may reduce the occurrence of this complication. In contrast, the safety of taking low doses of spironolactone was supported because the incidence of hyperkalemia was comparable to that of the standard treatment group. Furthermore, we considered that the interaction between spironolactone treatment and β-blockers or ACEI/ARB may primarily due to increased serum potassium levels after use of spironolactone in AMI with CKD patients. With the increased of serum potassium, the rate of adverse clinical outcomes in AMI with CKD population also increased. These findings underscore the importance of close monitoring of serum potassium levels with MRA treatment, particularly in CKD patients and in combination of β-blockers or ACEI/ARB patients, and carefully assessment of the risks and benefits for MRA in the context of elevated potassium levels.

### Study Limitations

This study was not a randomized blinded multicenter trial; thus, we cannot exclude residual confounds despite performing extensive adjustments. The analysis should be considered as only exploratory and further prospective trials were needed. Furthermore, the number of patients in high dose group was relatively small, and the results should be interpreted with caution. At last, we paid less attention to other side effects of spironolactone such as gynecomastia or other sex-hormones related effects.

## Conclusion

This study showed that using spironolactone may substantially reduce the risk of both all-cause mortality and re-admission in patients with AMI and CKD; low-dose spironolactone ( < 40 mg per day) was found to have the best effect and safety. We consider that spironolactone may be a useful therapy for improving the currently poor prognosis of patients with AMI and CKD. However, this was a relatively small sample size, single center, observational, retrospective, registry based clinical study and further prospective evaluation in adequately powered randomized trials were needed before further use of spironolactone in AMI with CKD population.

## Data Availability

The raw data supporting the conclusions of this article will be made available by the authors, without undue reservation.

## Abbreviations

CKD, chronic kidney disease; AMI, acute myocardial infarction; ACEIs, angiotensin-converting enzyme inhibitors; ARBs, angiotensin receptor blockers; MRA, mineralocorticoid receptor antagonists; PCI, percutaneous coronary intervention; eGFR, estimated glomerular filtration rate; BNP, brain natriuretic peptide; cTnI, troponin I; LVEF, left ventricular ejection fraction; HFpEF, heart failure with preserved ejection fraction.
